# Spatial analysis of campylobacter infection in the Canadian province of Manitoba

**DOI:** 10.1186/1476-072X-5-2

**Published:** 2006-01-16

**Authors:** Chris G Green, Dennis O Krause, John L Wylie

**Affiliations:** 1 Public Health Branch, Manitoba Health, Room 4050, 300 Carlton St., Winnipeg, Manitoba, R3B 3M9, Canada; 2 Department of Community Health, Faculty of Medicine, University of Manitoba, S111 - 750 Bannatyne Avenue, Winnipeg, Manitoba, R3E 0W3, Canada; 3 Department of Animal Science, Faculty of Agriculture and Food Sciences, University of Manitoba, Winnipeg, Manitoba, R3T 2N2, Canada; 4 Department of Medical Microbiology and Infectious Diseases, Faculty of Medicine, University of Manitoba, 750 Bannatyne Ave., Winnipeg, Manitoba, R3E0W3, Canada; 5 Cadham Provincial Laboratory, Manitoba Health, 750 William Ave., Winnipeg, Manitoba, R3E 3J7, Canada

## Abstract

**Background:**

The study describes population level variations in campylobacter incidence within the Canadian province of Manitoba, and the relationship to sociodemographic and landscape related characteristics. Using data derived from the Manitoba Health Public Health Branch communicable disease surveillance database, the study applied a number of spatial and ecological techniques to visualize, explore and model campylobacter incidence for the years 1996 to 2004. Analytical techniques used in the study included spatial smoothing, the spatial scan statistic, the Gini coefficient, and Poisson regression analysis.

**Results:**

The study demonstrated marked and statistically significant geographic variability in the rates of campylobacter incidence in Manitoba.. The incidence of campylobacter was observed to be significantly higher in populations living in rural and agricultural areas of the province, with the highest rates occurring in populations living in proximity to high densities of farm animals (cows, pigs, chickens). The study also observed that the age specific pattern of campylobacter incidence in rural Manitoba was very different than the urban pattern, with the incidence rate in the 0–4 year age group seven times higher in rural Manitoba than in the City of Winnipeg.

**Conclusion:**

The study demonstrates the value of a deploying a diverse set of spatial techniques to better understand the dynamics of an enteric disease such as campylobacter infection. The study concludes that there may be three distinct mechanisms for the transmission of campylobacter in Manitoba which are operating simultaneously. These include broad population exposure to a centralized food system endemically infected with the campylobacter organism, exposure to local level factors such as farm animals or contaminated water, and exposure to campylobacter infection through foreign travel.

## Background

Campylobacter infection is a leading cause of foodborne illness in western countries [1]. The disease, which results in acute enteritis of variable severity, is characterized by diarrhea, which is often bloody, as well as abdominal pain, malaise, nausea, and occasionally vomiting [1]. Risk factors associated with campylobacter infection include the consumption of various foods including unpasteurized milk [2-5], raw seafood and undercooked poultry [6-8]; contact with contaminated surface water [9]; contact with domesticated animals (puppies and kittens) [5,10]; contact with various farm animals including chickens, pigs and cows [11-13]; previous antibiotic use [14]; and foreign travel[15,16].

Few studies to date have used population based data to describe patterns and trends in campylobacter infection [1,17]. Most published studies have been either case control studies of small population sub-sets, or have drawn upon data obtained through sentinel surveillance systems. It appears that population based data on campylobacter infection is not generally available since there are few jurisdictions that have centralized public health surveillance systems with complete population coverage. Population based studies of campylobacter infection are critically required in order to better understand its temporal and spatial dynamics and its human and environmental determinants.

The current study draws upon the centralized Manitoba Health Public Health Communicable Disease database (MPHCDD) which collects information on all laboratory confirmed cases of campylobacter infection occurring in the province of Manitoba, Canada. The study describes the population level variations in campylobacter infection in Manitoba; the socio-demographic and environmental factors associated with these variations; and the implications of the geographic concentration of campylobacter infection for known disease etiology and for public health prevention efforts.

## Results

### Temporal and demographic trends

**Figure 1:**
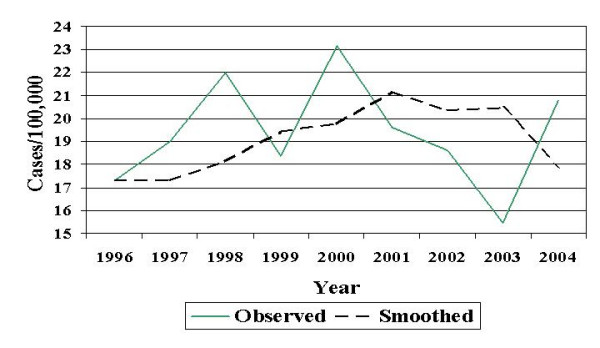
Campylobacter incidence, Manitoba, 1996 – 2004, time trend, cases/100,000.

Between 1996 and 2004 there were 1983 incident cases of campylobacter infection recorded, with an annual crude rate of 19.19 cases/100,000. Figure 1 shows annual campylobacter incidence from 1996 to 2004 and the smoothed trend line generated using the moving average filter. As illustrated, campylobacter incidence was relatively stable over the study period, increasing slightly between 1996 and 2001, and then decreasing again by 2004. Campylobacter incidence exhibited clear seasonal trends between 1996 and 2004, with the highest number of cases occurring in the summer (n = 680) and fall (n = 539) and the lowest number of cases occurring in the spring (n = 431) and winter (n = 333).

**Figure 2:**
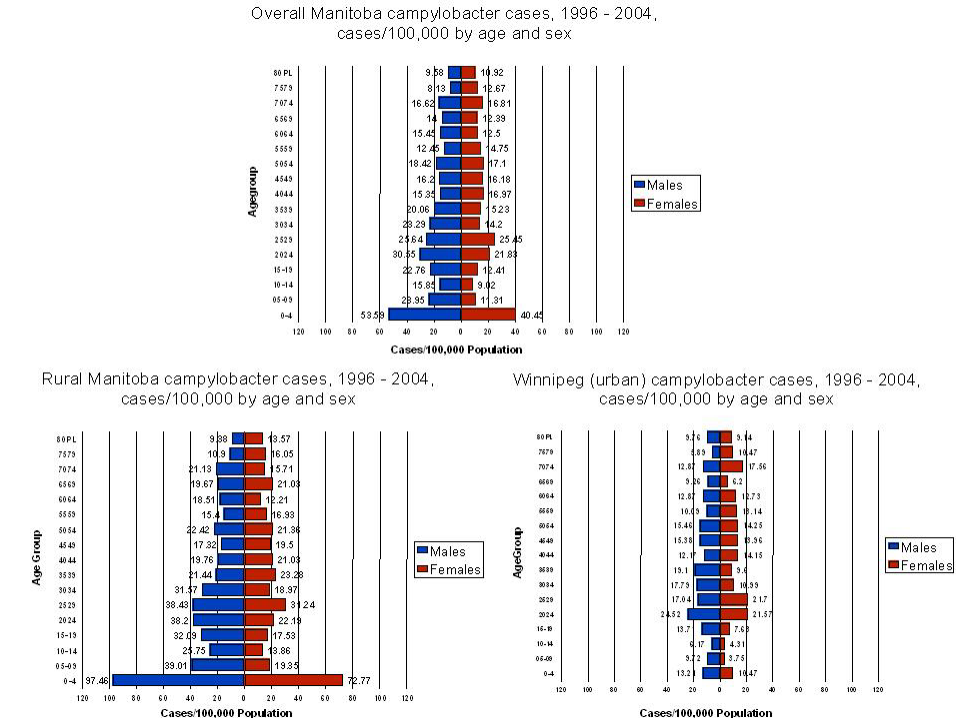
Campylobacter incident cases, Manitoba, 1996–2004, rates by age and gender, all of Manitoba vs. urban vs. rural.

For Manitoba as a whole, the highest rate of campylobacter infection was observed to occur in the 0–4 and 20–39 year age groups, with slightly higher rates occurring in males as compared to females (Figure 2). Large differences in the age structure of campylobacter incidence were observed, however, between Winnipeg (urban) and rural Manitoba. In rural Manitoba campylobacter incidence rates were higher in almost every age and gender category, with the greatest difference observed in the 0–4 year age group where rates in rural males were 7.3 times higher than in urban males (97.5 cases/100,000 vs. 13.2 cases/100,000), and 6.95 times higher in rural females as compared to urban females (72.8 cases/100,000 vs. 10.5 cases/100,000).

### Data visualization

**Figure 3:**
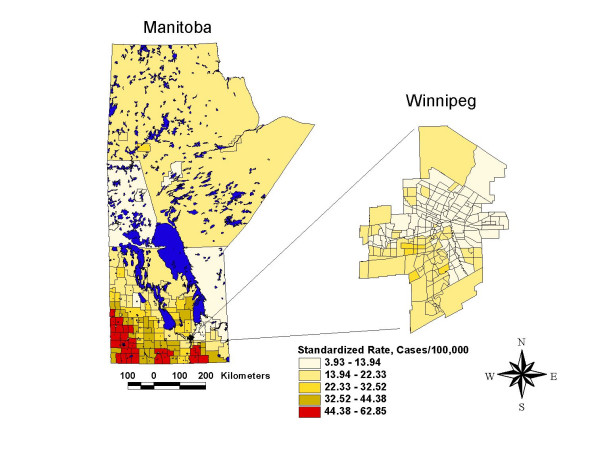
Campylobacter incidence map, Manitoba, 1996–2004, smoothed, age standardized to the 2001 general Manitoba population.

The thematic map of campylobacter incidence (Figure 3) shows marked geographic variability in the rate of campylobacter across the province of Manitoba. Regionally, the rates of campylobacter incidence range from less than 13.94 cases/100,000 in the central and northern core of the City of Winnipeg and in rural regions in the northwest and east central districts of the province, to greater than 44.38 cases/100,000 in southern rural areas of the province.

**Figure 4:**
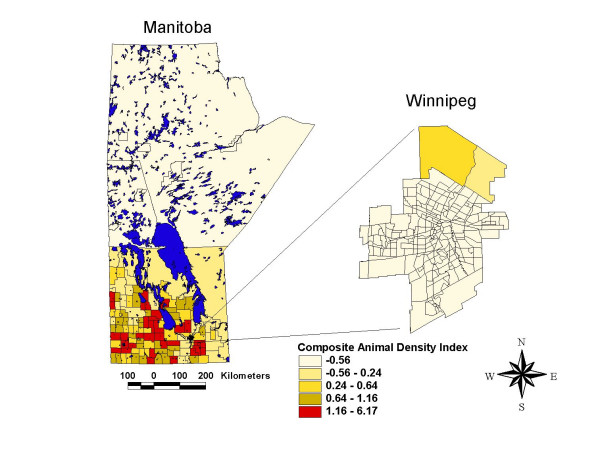
Composite animal density index (CADI) map, Manitoba, 2001.

Similarly, the thematic map of the composite animal density index (CADI) illustrated in Figure 4 shows marked geographic variability in animal densities across the province, with the highest composite animal density appearing in the same general areas as those observed for high campylobacter incidence.

### Data exploration

**Figure 5:**
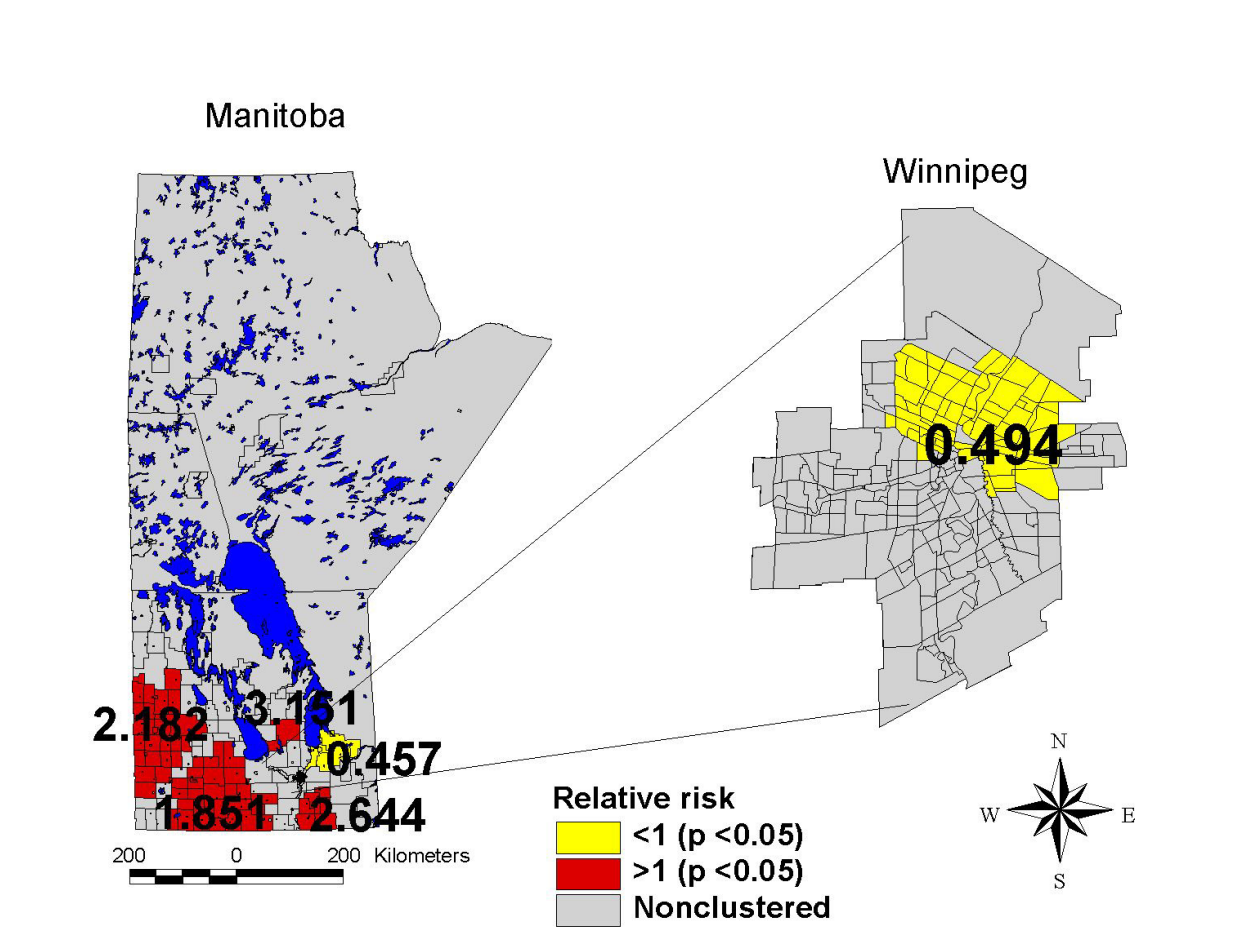
Campylobacter incidence analysis map, Manitoba, 1996–2004, using the spatial scan statistic, maximum cluster set at 10% of the study population.

The spatial scan statistic (Figure 5) identified statistically significant (p < 0.05) high and low rate clusters of campylobacter incidence in the same geographic areas as were identified in the data visualization stage (Figure 3). High rate clusters of campylobacter incidence with relative rates greater than 1 (ranging from 1.85 to 3.151) were identified in southern Manitoba, and low rate clusters with relative rates less than one (ranging from 0.494 to 0.457) were identified in the northern core of the City of Winnipeg and in southeastern Manitoba. This analysis confirms the non-random clustering of campylobacter disease events and suggests that the observed spatial patterns are unlikely to have occurred by chance alone.

**Figure 6:**
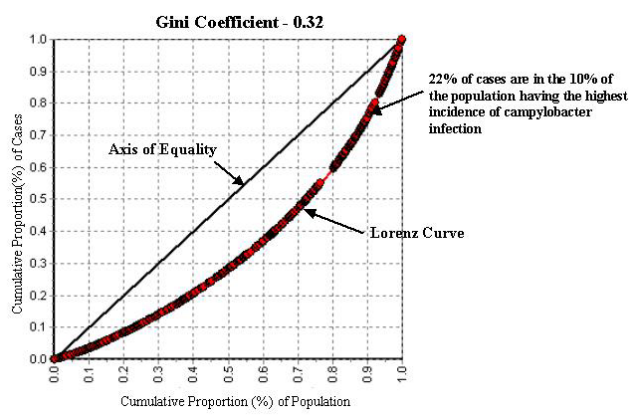
Gini coefficient, campylobacter incidence, Manitoba, 1996–2004.

The Gini coefficient for campylobacter incidence from 1996 – 2004 was 0.32, indicating a moderate level of geographic inequality in campylobacter incidence across geographic areas in Manitoba (Figure 6). The Lorenz curve used to calculate the Gini coefficient plots the ranked cumulative proportion of campylobacter incident cases against the ranked cumulative proportion of the population at risk Since cumulative proportion data was ranked in ascending order by the campylobacter incidence for each geographic area, those geographic areas experiencing the highest campylobacter incidence appear towards the top right of the Lorenz chart, while geographic areas having the lowest campylobacter incidence appear towards the bottom left of the Lorenz chart. The Gini coefficient is calculated as the proportion of the area under the axis of equality encompassed by the Lorenz curve. If campylobacter cases were equally distributed among geographic areas in Manitoba in proportion to the population at risk, the Lorenz curve would follow the 45 degree axis of equality exactly and the Gini coefficient would have a value of zero. The moderate deflection of the Lorenz curve downwards from the axis of equality suggests that campylobacter cases are not highly spatially concentrated in a small number of geographic areas in Manitoba; rather campylobacter cases are spread out quite evenly relative to the underlying population at risk (e.g. reading from the curve, the 10% of the population living in the geographic areas of the province having the highest campylobacter incidence contain only 22% of the campylobacter cases).

### Data modeling

Poisson regression analysis shows that campylobacter incidence varies by a range of socio-demographic and landscape factors. Table 1 shows the results of both the partially and fully adjusted poisson regression analyses in terms of rate ratios. A rate ratio is the ratio of the incidence rate in the category of interest compared to the rate in the reference category. The results of the partially adjusted model which describe the broad population and landscape characteristics associated with campylobacter incidence, indicate that camyplobacter incidence was the highest (significant rate ratios greater than 1) in the 0–19 year age group, in males, in rural areas, in populations most heavily involved in agricultural occupations, and in geographic areas with the highest densities of animals (CADI, cows, chickens and pigs). Socioeconomic status (SESI) was not related to campylobacter incidence in this model.

**Table 1:** Poisson regression analysis, campylobacter incidence, Manitoba, 1996 – 2004

	**Partially Adjusted Model**	**Fully Adjusted Model**
	Rate Ratio	Rate Ratio
^a^Agegroup:		
0–19	1.84 (1.48 2.29)	1.81 (1.48 2.20)
20–39	1.72 (1.39 2.14)	1.80 (1.47 2.19)
40–59	1.27 (1.01 1.59)	1.28 (1.04 1.58)
60–69	1.07 (0.80 1.44)	1.06 (0.81 1.38)
70+ (reference)	1.0	1.0
^a^Gender:		
Male	1.29 (1.16 1.43)	1.25 (1.14 1.38)
Female (reference)	1.0	1.0
^ab^Urban/Rural:		
Rural	2.06 (1.86 2.27)	1.46 (1.23 1.73)
Urban (reference)	1.0	1.0
^ab ^Socio-Economic Status Index (SESI)		
Low – Reference	1.0	1.0
Medium	1.03 (0.92 1.16)	1.14 (1.02 1.27)
High	0.86 (0.74 1.00)	1.49 (1.27 1.74)
^ab ^Agricultural Occupation		
Low – Reference	1.0	1.0
Medium	2.48 (2.23 2.76)	1.81 (1.59 2.06)
High	2.90 (2.43 3.45)	2.21 (1.82 2.69)
^ab ^Composite Animal Density Index (CADI)		
Low – Reference	1.0	1.0
Medium	1.96 (1.76 2.18)	1.15 (0.98 1.35)
High	2.81 (2.42 3.27)	1.68 (1.39 2.02)
^bc ^Cow Density		
Low – Reference	1.0	1.0
Medium	1.83 (1.63 2.05)	1.11 (0.95 1.31)
High	2.75 (2.41 3.13)	1.54 (1.30 1.83)
^bc ^Pig Density		
Low – Reference	1.0	1.0
Medium	1.86 (1.64 2.11)	1.12 (0.98 1.28)
High	2.73 (2.19 3.40)	1.99 (1.61 2.46)
^bc ^Chicken Density		
Low – Reference	1.0	1.0
Medium	1.21 (0.87 1.67)	0.91 (0.68 1.23)
High	2.58 (2.08 3.19)	2.11 (1.73 2.58)

^a ^Adjusted for all variables except cow, pig and chicken densities in the fully adjusted model

^b ^Adjusted for age group and gender in partially adjusted model

^c ^Adjusted for age group, gender, urban/rural, socio-economic status and agricultural occupation in fully adjusted model

( ) 95% Confidence Intervals, adjusted for model overdispersion

The fully adjusted model (Table 1) shows that all measures of animal density persist as significant predictors of campylobacter incidence (with significant rate ratios greater than 1) after controlling for the other sociodemographic and landscape variables in the model. In the fully adjusted model, soci-economic status (SESI) became a significant predictor, with campylobacter incidence increasing positively with increased socio-economic status.

**Figure 7:**
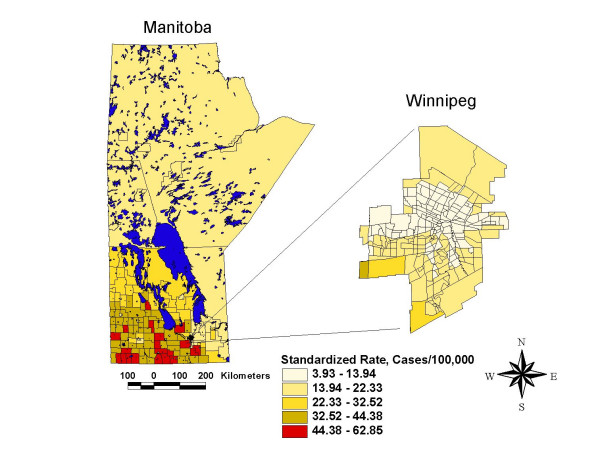
Predictive map of campylobacter incidence, Manitoba, 1996–2004, using predictions from fully adjusted poisson regression model.

A comparison of the map of actual campylobacter incidence rates (Figure 3) and the map of predicted campylobacter incidence rates (Figure 7) revealed a very similar spatial patterning of campylobacter incidence, indicating that the fully adjusted poisson model using CADI performed very well in predicting campylobacter incidence rates at the geographical level. The goodness of fit between predictions from the poisson regression model and actual observed rates of campylobacter incidence was further confirmed by a Spearman's rank correlation coefficient of 0.79 (p < .05) and a Pearson's R correlation coefficient of 0.83 (p < .05).

## Discussion

This study has described the temporal, spatial, and socio-demographic trends in campylobacter infection in Manitoba. Although significant seasonal fluctuations in disease incidence occurred between 1996 and 2004, campylobacter incidence remained relatively constant over the study period. Campylobacter incidence rates reported in this study are comparable to rates reported through the Food Net sentinel surveillance system in the U.S. [17] but generally lower than incidence rates reported for Canada and several of its provinces [41]. Whether the lower incidence in Manitoba vs. other provinces is real or related to diagnostic or reporting differences is unknown at this time. The pronounced geographic variation of campylobacter incidence associated with agricultural animal density observed in this is consistent with a recent Ontario study which used similar spatial approaches to study the geographic distribution of giardia, another common enteric infection thought to be associated with agricultural animal density [35]. The Ontario study, using both spatial smoothing and the spatial scan statistic, found marked spatial patterning in giardia incidence moderately associated with agricultural animal density and manure spreading.

We hypothesize three distinct mechanisms to account for the spatial patterns of campylobacter in Manitoba. First, the majority of cases of campylobacter cases may be the result of broad population exposure to a centralized food system endemically infected with the campylobacter agent. A number of studies have shown an almost universal contamination of commercial meat products with the campylobacter organism [42-44]. The low Gini coefficient of 0.32 observed in this study suggests that most cases of the disease are occurring in the general population living in low incidence geographic areas. As described, only 22% of campylobacter cases occurred in the 10% of the population living in the highest incidence areas for the disease. This suggests that while there may be local level factors which can dramatically increase the risk of infection, the majority of cases of campylobacter infection are the result of factors affecting all population groups (i.e. the food system). The implication of this for public health prevention efforts is that in order to significantly decrease the population incidence of an enteric disease such as campylobacter, public health efforts would have to focus on factors affecting the population as a whole; focusing only on high risk groups would have a limited effect on the overall population level incidence of the disease [45-47].

Secondly, the study results clearly show local level factors are strongly associated with increased risk of campylobacter infection. The partially adjusted regression model suggests that population groups living in rural areas, employed in agricultural occupations and living in geographic areas with exposure to high levels of agricultural animal densities have rates of campylobacter incidence two to almost three times higher than in lower risk areas of the province. Most importantly, the fully adjusted model confirms that exposure to high animal densities continues to be strongly associated with increased risk of campylobacter incidence once all other predictor variables have been controlled for. There may be other local factors broadly associated with living in rural agricultural areas of rural Manitoba which confer increased risk for campylobacter infection above and beyond the effects of animal density (e.g. consumption of unpasteurized milk, non-centralized and contaminated drinking water supplies). There is significant urgency in studying the etiological effects of local level factors in rural Manitoba given the extreme bias in rural Manitoba towards younger age groups in campylobacter incidence. As illustrated in Figure 2, the age structure of campylobacter infection in rural areas resembles infection patterns found in many developing countries [1], with age specific attack rates in the 0–4 year age group greatly exceeding those in older age groups.

Thirdly, the positive association of campylobacter incidence with socioeconomic status (observed in the fully adjusted regression model) suggests that once all other predictors have been controlled for, factors associated with high socio-economic status confer increased risk to the disease. We speculate that these factors could be related to increased opportunities for foreign travel to countries where campylobacter infection is endemic, consumption of luxury foods (such as raw seafood) or more frequent restaurant meals [6,15]. It would be important to confirm these hypotheses through further studies which collect information on the specific causal pathways which may be operative in higher SES groups (dietary and travel behavior, genotype analysis). This study has a number of methodological limitations that must be kept in mind when interpreting its results. First, the data modeling component of the study is based upon an ecological design. This means that the measures of socio-economic status, occupation, and animal exposure were measured at the geographical level and cannot be considered direct properties of individuals [48]. This design was adopted since the socio-demographic and ecological data required for the study (other than age and sex) were simply not available on the individual health record. It is important to note that in this study the ecological estimates that were applied to individual cases of campylobacter infection were generated from a set of spatial units with an average population size of only 2000 persons, minimizing the possibility of significant heterogeneity within those geographic areas biasing individual level sociodemographic estimates [49].

Secondly, the Poisson regression modeling that was employed in this study to examine the relationships between campylobacter incidence and its predictors generated only global measures of association. It is doubtful that the relationship between campylobacter infection and its predictors are constant across all regions and global statistical measures of association may tend to render invisible local heterogeneities of significant importance. Techniques such as the multivariate LISA statistic [50,51] and geographically weighted regression [52], with applicable software for the calculation have only recently become available. The use of local measures of association warrant application to the study campylobacter in Manitoba, but are beyond the scope of the current study.

Thirdly, the spatial smoothing and spatial scan techniques that were used to visualize and confirm the location of high and low rate areas of campylobacter incidence have the potential to generate results that are artefacts of the methods themselves. Both the adaptive mean nearest neighbor smoothing technique and the spatial scan statistic used in this study combine data from contiguous geographic areas in order to create stable rate estimates for local geographic areas. These approaches are based upon the assumption that contiguous geographic areas have similar characteristics, an assumption that may sometimes be violated. Both of these techniques may also suffer from edge effect biases which occur when rate estimates are made for geographic areas at the borders of the study area [53]. However, the fact that these two techniques (which used somewhat different methods to estimate local disease rates) generated similar visual spatial patterns in campylobacter incidence (Figures 3 and 5), provides confidence that the local disease patterns identified in this study are indeed real.

Fouthly, campylobacter incidence rate estimates are most certainly under-estimated in this study. Previous studies have estimated that only one out of thirty eight cases of campylobacter are actually reported through public health surveillance systems [54]. This suggests that perhaps there were as many as 75,354 cases of campylobacter infection occurring between 1996 and 2004 (as compared to 1983 reported cases). What is not known is whether there are systematic differences across the province in the rates of reporting campylobacter infections to public health authorities which may be affecting the spatial and demographic patterns observed in this study.

Finally the use of postal code alone to geocode campylobacter cases most certainly resulted in a number of errors in allocating disease cases to the small geographic areas used in this study. This is because postal codes in the Canadian context have been designed for mail delivery, not for the allocation of health events. In rural areas this is a much greater problem since a postal code is often associated with a mail delivery route or a rural postal office location which serves a large geographic area that can cut across municipal boundaries; in urban areas the use of postal code alone results in fewer errors since a single postal code is usually associated with residences on a single block face or served by a super mail box. Compounding this problem is the fact that postal codes are periodically retired and then "re-born" (usually in the same geographic area), making the use of the latest postal code conversion file problematic when used with historical data. In this study the geocoding problem is an intractable one (i.e. a more precise geographic location cannot be obtained for disease cases) since postal code is the only reliable geographic identifier that exists on health records in the MPHCDD. However, we would argue that the spatial methods used in this study may be relatively robust to these geocoding errors in the following ways. First, the same geocoding method was applied to both the numerator and denominator in order to ensure that geocoding errors at the geographic level altered the size of the numerator and the denominator in the same general direction. Secondly, both the spatial smoothing and the spatial scan statistic techniques used in this study aggregate data in small contiguous geographic areas, thereby minimizing the effect of small area mis-classification on small area rate estimation and cluster identification. Thirdly, the Poisson regression analysis approach used in this study, which aggregates geographic areas having similar ecological characteristics (essentially a form of virtual smoothing) also minimizes the effect of small area mis-classification since it is likely that many "mis-classified" events would end up in the same ecological quantile regardless of whether they were in their true location or in the location estimated in this study. In future studies, where both postal code and a more precise geographic identifier are available, it would be important and useful to undertake a sensitivity analysis to assess how robust these spatial methods actually are to different geocoding methods.

## Conclusion

The results of this study suggest that observed patterns of campylobacter incidence may be the result of a complex interplay of factors operating at both global and local scales of influence. Campylobacter infection may be occurring in different places for different reasons. Further research is required to identify and confirm specific causal pathways that can explain infection patterns. Future investigations should examine whether particular genotypes predominate in urban vs. rural areas – i.e. are urban cases more related to genotypes originating from foreign travel and food sources, and to examine if there is any correspondence between the genotypes affecting both local animal and human populations.

## Methods

### Study setting

The study was conducted in the central Canadian province of Manitoba. Manitoba has a population of 1.1 million people with more than one half (645,000) residing in the City of Winnipeg, the provincial capital. The majority of Manitobans are of European descent whereas 10% of the population is self-identified as having Aboriginal ancestry. Manitoba has a provincial health insurance plan, and all residents of the province are eligible to receive health care services with no payments required. It is generally assumed that 99.9% of all residents of Manitoba are registered under the health insurance plan since registration is a pre-requisite to receiving any kind of insured health service.

### Data sources

All data sources used in the study were aggregated/geocoded to the standard set of geographic areas used by Manitoba Health for research and surveillance. Within the City of Winnipeg these were local neighborhoods (n = 230) and in rural areas these were health municipalities (n = 268) which are synonymous with Statistics Canada's census sub-divisions. The resulting set of 498 geographic areas had an average population size of 2000 and ranged in size from less than 100 (industrial park areas and newly established neighborhoods in the City of Winnipeg; First Nation communities in rural Manitoba) to a high of 41,500 (City of Brandon in rural Manitoba).

Campylobacter case data for the years 1996 to 2004 were extracted from the Manitoba Health Public Health Communicable Disease database (MPHCDD) using the ICD9 code 008.43. The MPHCDD, as an integrated component of Manitoba's universal health care system, has complete population coverage and contains all cases of campylobacter infection reported to the provincial public health system. The MPHCDD was implemented in 1996 and pre-1996 data was not available for analysis in a comparable format.

In Manitoba, a confirmed case of Campylobacter is considered to be the isolation of *Campylobacter jejuni *or *C. coli *from any site, regardless of symptoms. Campylobacter cases, under the Public Health Act of Manitoba, are reportable by all laboratories in the province. Although there are exceptions, most of these laboratories restrict reporting to *C. jejuni *or *C. coli *only, as per the case definition. Therefore, although a small percentage of cases in the MPHCDD would be other species of *Campylobacter*, the majority would be *C. jejuni *or *C. coli*.

Laboratory diagnostic performance for all facilities in Manitoba is overseen by the Manitoba Quality Assurance Program (MANQAP) and other national and international programs. As a result of these quality assurance programs, we expect that the variations in incidence that we identified would represent real differences in incidence rather than differences in the diagnostic ability of laboratories in different parts of the province.

The ecological measure of socio-economic status used in the study was calculated by combining small area estimates (rural health municipalities and Winnipeg neighborhoods) from custom tabulations derived from the 2001 Census Canada micro data files [18] for average family income, the unemployment rate, and the percentage of the adult population having some post-secondary education. The resulting composite index known as the socio-economic status index (SESI) was calculated using the SigEpi program [19] with standardized Z scores and equal weights attributed to each variable. The ecological measure of the percentage of the labour force involved in an agricultural occupation was also obtained from the 2001 Canada Census micro data files [18].

Animal density data for pigs, cows and chickens by census sub-division were obtained from the 2001 Canadian Census of Agriculture [20] and the number of animals per square kilometer in each rural health municipality of the province was calculated. Larger rural towns (which are classified as health municipalities) were classified with the animal density estimates of the average of their contiguous geographic neighbors. An overall measure of animal density was created by combining small area estimates of pig, cow and chickens densities. The resulting composite animal density index (CADI) was calculated using the SigEpi program [19] with standard Z scores, with differential weights attributed to each animal type. The weights attributed to each animal type were based upon published animal units [21], a measure widely used to estimate the feed inputs and manure outputs of farm animals. In the calculation of CADI, cows were weighted 71%, pigs 28% and chickens 1%.

Population denominator data from 1996 to 2004 were extracted electronically from the Manitoba Health Population Registry. Urban residence was assigned to those individuals living in the city of Winnipeg, the only urban center in Manitoba with a population greater than 50,000.

### Assessment of temporal and demographic trends

To assess temporal trends in campylobacter disease rates in Manitoba, annual incidence rates were calculated for the years 1996 to 2004. Annual incidence rates for 1996 to 2004 were calculated using the mid-year population at risk, based on the Manitoba population registry. To remove extreme fluctuations in annual campylobacter incidence rates (due to a small number of annual events), annual incidence rates were smoothed using a moving average filter in Stata 8.0 [22]. In addition to overall population annual rates, the data was further stratified by season and by age and gender.

### Spatial methods

Both Campylobacter cases and population denominator data were geocoded to neighborhoods in the City of Winnipeg and to health municipalities in the rural areas of Manitoba based on postal code using the 2004 postal code conversion file supplied by Statistic Canada [23]. Where one postal code related to more than one geographic area, the single link indicator on the postal code conversion file was used to allocate the record.

The spatial analysis of the data was undertaken in three steps according to the framework for spatial analysis proposed by Gatrell and Bailey [24]. The three stages of the framework are data visualization (visual depiction of the data spatially in the form of maps), data exploration (identifying the relevant statistical properties of the data), and data modeling (modeling the statistical relationships between predictor and outcome variables across space). In the first step, data visualization, directly standardized incidence rates for the years 1996–2004 combined were estimated for each of 498 geographic areas used in the study. In order to control for unstable rate estimates resulting from small case counts, stable rate estimates for campylobacter were generated using an adaptive mean nearest neighbor smoothing algorithm [25,26] that was implemented using a program written in Epi Info 6.04d [27]. Geographic areas with a population size of 10,000 or more in 1998 were not smoothed; geographic areas with a population size of less than 10,000 were smoothed by adaptively borrowing both numerator and denominator data from neighboring geographic areas to the degree required to generate a denominator of approximately constant size (10,000 persons). The spatial contiguity file required for the spatial smoothing calculations contained information on first, second and third order neighbors and was created using S-Plus for Arc-View [28]using population weights from the year 1998. Directly standardized rates using the 2001 general Manitoba population at risk (as estimated from the Manitoba Population Health registry) as the standard were then calculated using the data borrowed in the smoothing process. Arc-View 3.2 [29] was used to generate a thematic map of campylobacter incidence for Manitoba. In order to facilitate a visual comparison of campylobacter incidence with overall animal density, a thematic map of CADI for Manitoba was also generated.

In the second step, the spatial scan statistic was used to confirm that disease patterns identified through the data visualization stage were real and not due to random spatial variation. The spatial scan statistic, which identifies high and low rate cluster areas through the aggregation of numerator and denominator data of contiguous geographic regions was calculated using the Satscan 5.1 software [30]. The software was set to find age adjusted clusters with a maximum size of 10% of the study population, and detected clusters were tested for significance using 999 Monte Carlo random simulations. The program assumes a Poisson distribution and calculates indirectly standardized rates (expressed as the relative risk which is the observed rate divided by the expected rate) for each identified geographic cluster. Only clusters significant at the p < 0.05 level were retained for mapping. The specifics of the spatial scan statistic and the use of the Satscan software have been described previously [31,32]. The spatial scan statistic has been employed previously to study both chronic [33,34] and infectious [35-37] diseases.

The Gini coefficient, a measure of inequality ranging from 0 (absolute equality) to 1 (absolute inequality), was then calculated using the formula proposed by Castillo-Salgado et. al. [38] and was implemented using a program written for this purpose in Epi Info 6.04d [27]. The Gini coefficient was calculated from the ranked cumulative proportions of campylobacter cases and the population at risk at the level of the small geographic areas used in this study. Gini coefficient calculations were double checked using the EPIDAT 3.0 software package distributed by the Pan American Health organization [39].

In the third step, data modeling, the socio-demographic and landscape characteristics associated with each of the 498 geographic areas study were classified into tertiles using the Jenks natural breaks algorithm using Arc-View 3.2 [29]. These variables included SESI, the percentage of the labour force involved in an agricultural occupation, CADI, and individual animal densities (cows, pigs, chickens). These were then assigned to individual records in the database. '

In order to describe the broad population and landscape characteristics associated with campylobacter incidence, the poisson regression functionality in NCSS 2001 [40] was used to generate age and gender adjusted rate ratios for each of the predictor variables using the population at risk (partially adjusted model). To determine if measures of animal density persisted as statistically significant predictors of campylobacter incidence after controlling for all other variables, a fully adjusted model was also implemented. In this model, all non-animal density predictor variables were entered into the regression model at the same time with each of the animal density predictors (CADI, cow density, pig density, and chicken density) entered separately. This was done to ensure that a potentially high level of covariance between the animal density predictors did not obscure the relationship between individual animal density measures and campylobacter incidence. Confidence intervals were corrected for model over-dispersion using the phi multiplier function in NCSS. In order to explore the geographic "goodness of fit" or performance of the fully adjusted model employing CADI as the animal density predictor, a predictive map was produced using the outputs from the regression model. Visual comparison between the map of actual campylobacter incidence rates and predicted incidence rates was undertaken. To more formally assess the strength of the relationship between the observed and predicted incidence rates at the geographic level, the Spearman's rank and Pearsons correlation statistics were calculated.

## List of Abbreviations

MPHCDD

Manitoba Public Health communicable disease database

MANQAP

Manitoba Quality Assurance Program

SESI

Socio-economic status index

CADI

Composite animal density index

## Competing interests

The authors of this paper do not have any commercial interests or other associations which pose a conflict of interest with this paper.

## Authors' contributions

CG was involved in the conceptualization, research design, execution and write up of the study manuscript. DK provided expert input into the use of animal density data and the implications for study design. JW was involved in the conceptualization of the study and provided expert input into the use of campylobacter surveillance data. All authors were involved in the preparation of the manuscript.
